# Impact of the COVID-19 Pandemic on Obesity, Metabolic Parameters and Clinical Values in the South Korean Adult Population

**DOI:** 10.3390/jcm13102814

**Published:** 2024-05-10

**Authors:** Anna Kim, Eun-yeob Kim, Jaeyoung Kim

**Affiliations:** 1 Department of Dermatology, College of Medicine, Korea University, Seoul 02841, Republic of Korea; annaykim31@gmail.com; 2 Research Institute for Skin Image, Korea University College of Medicine, Seoul 08308, Republic of Korea; key0227@korea.ac.kr; 3 Department of Convergence Medicine, College of Medicine, Korea University, Seoul 02841, Republic of Korea

**Keywords:** COVID-19 pandemic, obesity, metabolic parameters, clinical values, population health

## Abstract

This study aimed to evaluate the effects of the COVID-19 pandemic on obesity, metabolic parameters, and clinical values in the South Korean population. Data from the seventh and eighth National Health and Nutrition Examination Surveys were analyzed, comprising 3560 participants in 2018 (pre-COVID-19) and 3309 participants in 2021 (post-COVID-19). The study focused on adults aged 19 years and older who were overweight (BMI ≥ 25 kg/m^2^). The results showed a significant increase in waist circumference (approximately 2 cm), BMI (approximately 0.11 kg/m^2^), systolic blood pressure, fasting blood sugar (1.76 mg/dL higher), and glycated hemoglobin (0.14% higher) in the post-COVID-19 group compared to the pre-COVID-19 group. Additionally, the prevalence of hypercholesterolemia increased by 4% after the COVID-19 pandemic. These findings suggest an increased risk of obesity, abdominal obesity, and metabolic disorders, such as blood sugar disorders, in the post-COVID-19 period. Urine analysis revealed abnormal findings, including occult blood, urobilinogen, hematuria, proteinuria, ketone urea, glycosuria, and bacteriuria. The study highlights the negative impact of lifestyle changes, such as reduced physical activity and social gatherings, on physical vital signs and clinical values during the COVID-19 pandemic.

## 1. Introduction

The COVID-19 pandemic, also referred to as the “COVID-19 crisis”, had a sudden and massive impact on daily life, politics, economics, society, and culture [1,2]. However, three years after the global outbreak that originated in Wuhan, China, the incidence rate has decreased, but the pandemic has not yet ended [3]. The most significant changes resulting from this health crisis were not limited to the healthcare sector alone; the event led to a restructuring of the socioeconomic order and a fundamental transformation of the social system [4]. Due to the outbreak of the novel infectious disease COVID-19, human physiology has undergone many alterations [1,4]. Additionally, efforts have been made to prevent the spread of the infectious disease by imposing restrictions on global transportation [5]. Furthermore, social and personal activities have been forcibly reduced and prohibited [6,7]. Unlike previous outbreaks, such as those of SARS and MERS, COVID-19 has had a significant impact on politics, economics, society, and culture [8]. The Ministry of Culture, Sports, and Tourism in South Korea conducted a survey in 2020 on the public’s perception of COVID-19 quarantine measures. The survey revealed that 84.3% of respondents took the situation seriously, while 55.8% expressed concern and worry about the possibility of infection [9].

In 2018, the Korean Diabetes Association (KDA) estimated that between 13.8% and 26.9% of adults over the age of 30 in South Korea are expected to have impaired fasting glucose, and that 35% of diabetic patients have not yet been diagnosed with diabetes [10]. South Korea is experiencing a significant prevalence of diabetes, with 53.2% of individuals with diabetes also having hypertension, and 72% having hypercholesterolemia, necessitating urgent and systematic management strategies for this patient population [10,11,12].

In this study, we investigated the alterations in physical vital signs, blood tests, and urine tests among overweight and obese adults aged 19 years and above in South Korea, before and after the onset of the COVID-19 pandemic. To achieve our objective, we aimed to examine the variations in physical vital signs, blood tests, and urine test values from the data collected by national institutions. Additionally, we aimed to assess how the risk of developing adverse health conditions changed. Based on these findings, we intended to provide a foundation for research aimed at identifying, managing, and preventing factors that can have adverse effects on both mental and physical health during and after the COVID-19 pandemic. Furthermore, we believe that this research will provide essential data for managing the health of individuals in the event of future infectious disease outbreaks, similar to that of COVID-19.

## 2. Materials and Methods

### 2.1. Study Designs and Sampling

**Figure 1:**
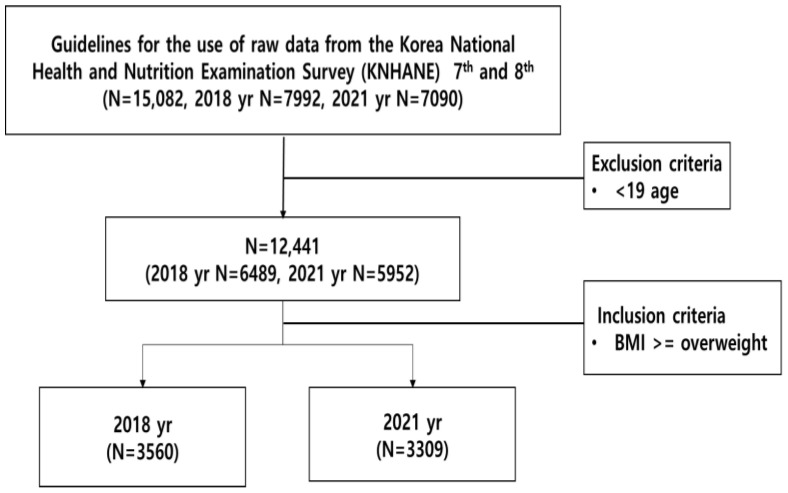
Data cleaning process flow. (The year 2018 was a year before the COVID-19 outbreak, and 2021 was the year after the COVID-19 outbreak.)

This study utilized raw public data from the seventh and eighth National Health and Nutrition Examination Surveys, which are nationally approved statistical sources (No. 117002), for the second time. Out of a total of 15,082 participants, only individuals with a body mass index (BMI) classified as overweight or obese (BMI ≥ 25) and aged 19 years or older were included in the study. Participants under the age of 19 were excluded. The final study sample comprised 3560 participants from 2018 and 3309 participants from 2021. Data were obtained through the online distribution procedure of the relevant institution, utilizing national open data. The two groups were designated as pre-COVID-19 and post-COVID-19, respectively. The study focused on overweight and obese adults aged 19 years and older, with a BMI of 25 or higher (Figure 1).

### 2.2. Data Variables

The variables examined in this study include the following: (1) physical characteristics, such as gender, age, height, weight, waist circumference, body mass index (BMI) based on Asian standards, pulse rate, and blood pressure (average of two measurements); (2) blood test parameters, including fasting blood glucose, glycated hemoglobin (HbA1c), total cholesterol, high-density lipoprotein (HDL) cholesterol, triglycerides, low-density lipoprotein (LDL) cholesterol, the prevalence of hypercholesterolemia, the prevalence of hypertriglyceridemia, aspartate aminotransferase (AST), alanine aminotransferase (ALT), hepatitis B surface antigen positivity, hepatitis C antibody positivity, hemoglobin, hematocrit, the prevalence of anemia, blood urea nitrogen, serum creatinine, white blood cell (WBC) count, red blood cell (RBC) count, and platelet count; and (3) urine test parameters, including uric acid, urine pH, nitrite, urine protein, urine glucose, and urine ketone. Additionally, participants underwent tests for urine bilirubin, urine occult blood, urobilinogen, urine creatinine, and urine bilirubin, as well as measurements of sodium, potassium, and cotinine levels in urine.

### 2.3. Data Analysis

The study variables were categorized as being either categorical or continuous variables. Categorical variables were presented as frequencies and percentages, while continuous variables were reported as means and standard deviations after assessing the normality of their distributions. Differences between the groups were analyzed by using the Mann–Whitney U test for continuous variables and Fisher’s exact test or the chi-square test for categorical variables, as appropriate. Multiple regression analysis was performed to identify variables associated with the severity of the COVID-19 outbreak, with a significance level criterion of 0.10 for input variable selection. All statistical analyses were conducted using IBM SPSS software (version 25.0, IBM Corp., Armonk, NY, USA), with a significance level of 0.05.

## 3. Results

### 3.1. Blood Pressure Changes in Overweight/Obese Adults Pre-/Post-COVID-19

Changes in the general characteristics of the overweight group before and after the COVID-19 outbreak are presented in Table 1. A significant difference was observed in systolic blood pressure (122.12 ± 16.29 mmHg vs. 123.32 ± 15.12 mmHg) and diastolic blood pressure (77.39 ± 10.45 mmHg vs. 75.56 ± 9.60 mmHg) between the group classified as obese (BMI ≥ 30 kg/m^2^, 13.4%) and the non-obese group (BMI 25–29.9 kg/m^2^, 16.7%) (Table 1).

### 3.2. Blood Test Changes in Overweight/Obese Adults Pre-/Post-COVID-19

The differences in blood test values before and after the COVID-19 outbreak are presented in Table 2. Significant differences were observed in fasting blood glucose levels (104.84 ± 24.24 mg/dL vs. 106.60 ± 25.98 mg/dL), glycated hemoglobin (HbA1c) levels (5.83 ± 0.84% vs. 5.97 ± 0.90%), total cholesterol levels (193.81 ± 39.21 mg/dL vs. 189.07 ± 40.17 mg/dL), high-density lipoprotein (HDL) cholesterol levels (48.02 ± 11.26 mg/dL vs. 49.17 ± 11.66 mg/dL), and triglyceride levels (154.60 ± 118.50 mg/dL vs. 141.33 ± 106.48 mg/dL) between the pre-COVID-19 and post-COVID-19 groups (Table 2).

### 3.3. Urinalysis Changes in Overweight/Obese Adults Pre/Post-COVID-19

The results of the differences in urine test values before and after the COVID-19 outbreak are presented in Table 3. Significant differences were observed in the percentage of participants with negative results for urinary protein (80.2% vs. 90.9%), negative urinary glucose (94.7% vs. 91.9%), negative urinary ketone (98.5% vs. 98.9%), negative urinary bilirubin (99.3% vs. 100.0%), negative urinary occult blood (82.8% vs. 93.5%), negative urobilinogen (99.5% vs. 99.3%), as well as in urine creatinine levels (147.08 ± 80.10 mg/dL vs. 125.89 ± 74.77 mg/dL), urine sodium levels (116.79 ± 48.09 mmol/L vs. 113.66 ± 47.75 mmol/L), urine potassium levels (52.66 ± 23.22 mmol/L vs. 41.51 ± 20.75 mmol/L), and urine cotinine levels (346.22 ± 718.56 ng/mL vs. 783.69 ± 826.45 ng/mL) between the pre-COVID-19 and post-COVID-19 groups (Table 3).

### 3.4. Multivariate Analysis of Blood Test Changes Pre-/Post-COVID-19

The results of the multivariate analysis on changes in blood test values before and after the COVID-19 outbreak are presented in Table 4. The analysis revealed that systolic blood pressure was significantly higher after the COVID-19 outbreak (β = 0.964, 95% CI: 0.956–0.972, *p* < 0.001). The results of the study showed significant associations between various health indicators and the outcome variable. Specifically, diastolic blood pressure (β = 0.964, 95% CI: 0.956–0.972, *p* < 0.001), high-density lipoprotein (HDL) cholesterol (β = 1.010, 95% CI: 1.004–1.016, *p* = 0.001), hemoglobin (β = 0.280, 95% CI: 0.240–0.326, *p* < 0.001), hematocrit (β = 1.476, 95% CI: 1.386–1.572, *p* < 0.001), blood urea nitrogen (β = 0.963, 95% CI: 0.950–0.975, *p* < 0.001), red blood cell (RBC) count (β = 1.352, 95% CI: 1.016–1.801, *p* = 0.039), and platelet count (β = 0.996, 95% CI: 0.995–0.997, *p* < 0.001) were significantly associated with the outcome variable (Table 4).

### 3.5. Multivariate Analysis of Urine Test Changes Pre-/Post-COVID-19

The results of the multivariate analysis on the changes in urine test values before and after the COVID-19 outbreak are presented in Table 4. After the COVID-19 outbreak, compared to the pre-COVID-19 period, there was a significant increase in trace urinary protein levels (β = 0.385, 95% CI: 0.208–0.486, *p* < 0.001), urinary glucose levels of +++ or higher (β = 2.311, 95% CI: 1.350–3.955, *p* = 0.002), trace urinary occult blood levels (β = 0.374, 95% CI: 0.246–0.568, *p* < 0.001), urinary occult blood levels of + or higher (β = 0.203, 95% CI: 0.085–0.481, *p* < 0.001), urine creatinine levels (β = 1.003, 95% CI: 1.001–1.005, *p* < 0.001), urine potassium levels (β = 0.973, 95% CI: 0.968–0.977, *p* < 0.001), and urine cotinine levels (β = 1.001, 95% CI: 1.001–1.001, *p* < 0.001) (Table 4).

## 4. Discussion

This study analyzed changes in blood and urine test results among adults aged 19 years and older who were overweight before and after the outbreak of COVID-19, an emerging infectious disease. The COVID-19 pandemic imposed significant lifestyle changes and various restrictions on daily activities. It was deemed necessary to monitor the impact of these changes on physical health indicators.

This study found that participants’ mean waist circumference increased by approximately 2 cm, and that their mean body mass index (BMI) increased by approximately 0.11 compared to pre-pandemic measurements. Additionally, their mean systolic blood pressure was higher than pre-COVID-19 levels. An increase in blood pressure is known to correlate with a higher incidence of hypertension. Previous studies have reported an association between the prevalence of hypertension and the incidence of kidney disease. Research indicates that maintaining appropriate blood pressure levels can reduce the risk of kidney disease by an odds ratio (OR) of 0.42 [13].

The COVID-19 pandemic imposed limitations on daily activities and reduced in-person interactions, which may have negatively impacted physical well-being. Adolescence is a critical period for developing lifelong health habits, both mentally and physically [14,15,16,17]. It is noteworthy that individuals over 19 years of age made efforts to sustain their weight during the pandemic, as evidenced by this study. When assessing disease risk, there was an OR of 1.019 for increased systolic blood pressure, an OR of 0.964 for increased diastolic blood pressure, and an OR of 0.999 for increased high-density lipoprotein (HDL) cholesterol levels.

For diabetes, fasting blood glucose and glycated hemoglobin are recognized as important diagnostic factors [18,19]. In clinical practice, risk factors related to diabetes, such as genetics and lifestyle, are considered important, along with disease risk factors associated with obesity. In this study, the mean fasting blood glucose was 1.76 mg/dL higher, and mean glycated hemoglobin was 0.14% higher than pre-pandemic levels. The pandemic led to an increased risk of diabetes due to restrictions on physical activity and sudden lifestyle changes [18,20,21,22].

The prevalence of hypercholesterolemia has increased by 4% since the start of the COVID-19 pandemic. According to the 2010 National Health and Nutrition Examination Survey, the prevalence of hypercholesterolemia increased significantly from 8.0% in 2005 to 11.5% in 2009 among individuals aged 30 years and older [23]. In 2016, the prevalence rose to 14.4% among those over 30 years of age [24]. These findings suggest an ongoing increase in the prevalence of dyslipidemia. The present study revealed a mean increase of 0.38 mg/dL in high-density lipoprotein (HDL) cholesterol levels. Dyslipidemia is characterized by decreased HDL cholesterol concentration [25,26,27]. Low HDL cholesterol levels are specifically linked to cardiovascular disease risk. The Framingham Study by Gordon et al. (1977) found that, among adults aged 49–82 years, individuals with low HDL cholesterol levels had a higher incidence of cardiovascular disease, even with low low-density lipoprotein (LDL) cholesterol [28].

Additionally, low HDL cholesterol levels have been associated with abdominal adiposity [29], metabolic syndrome [30], cognitive impairment and dementia [31], impaired fasting glucose [32], and diabetes [33]. Therefore, managing obesity and daily lifestyle habits is crucial for maintaining healthy HDL cholesterol levels, which are strongly linked to adult diseases like cardiovascular disease [34,35]. The observed changes were likely due to decreased physical activity, the adoption of a Westernized diet, and pandemic-related lifestyle restrictions. These factors can lead to increased obesity, abdominal obesity, and a risk of conditions such as dysglycemia. In modern society, various home-based training programs can increase aerobic and resistance exercise, which is particularly important during periods of restricted movement due to infectious diseases like COVID-19. Appropriate physical activity can also aid weight management and reduce the risk of dysglycemia that may be exacerbated by frequent alcohol consumption. Furthermore, if hepatocellular injury is severe, aspartate aminotransferase (AST) levels rise more than those of alanine aminotransferase (ALT). When the hepatocyte membrane is damaged, both AST and ALT enzymes are released into the bloodstream, causing an increase [36]; however, elevated AST and ALT levels do not always indicate the extent of hepatocyte necrosis or injury. Levels exceeding five times the upper limit are considered indicative of impaired liver function [36]. In this study, mean AST levels increased by 1.18 U/L and mean ALT levels increased by 1.05 U/L compared to pre-pandemic levels.

Anemia is a condition that often coexists with various diseases and is particularly prevalent in chronic conditions such as infectious diseases, autoimmune disorders, chronic kidney disease, and cancer [37]. The prevalence of anemia was 3.9% higher in this study, with a mean decrease of 0.03 × 10^6^/μL in red blood cells and a mean decrease of 0.09 × 10^3^/μL in white blood cells. Anemia is characterized by a lower-than-normal number of red blood cells, which are responsible for oxygen transport, leading to an increased risk of tissue hypoxia. During a recent study on COVID-19, mean hemoglobin levels decreased by 0.34 g/dL. This decrease has been linked to hemoglobinopathies and hypoxic damage, potentially leading to the development of hypoxemic blood disorders due to dysregulated iron metabolism [38]. The emergence of novel infectious and inflammatory viral diseases poses unknown outbreak risks [39].

Urinalysis can detect abnormalities such as occult blood, urobilinogen, hematuria, proteinuria, ketonuria, glycosuria, and bacteriuria. It is primarily performed to detect and manage diseases of the kidneys and urinary tract [40]. In this study, the prevalence of proteinuria was 10.7%, and glycosuria was 2.8%. The prevalence of ketonuria was 0.4%, and urobilinogen was 0.7%. The prevalence of hematuria was 10.7%, while mean urobilinogen levels decreased by 0.2 mg/dL. Mean urea nitrogen decreased by 21.19 mg/dL, while mean sodium increased by 3.13 mmol/L. Mean potassium decreased by 11.51 mmol/L. The presence of proteinuria was significant, with an odds ratio (OR) of 0.385. Similarly, the presence of glycosuria and hematuria was significant, with ORs of 0.374 and 0.203 for trace and gross hematuria, respectively. Urinalysis indicated that mean hemoglobin was likely to decrease by 0.280 g/dL, while mean hematocrit was likely to increase by 1.746%. Blood urea nitrogen was likely to decrease by 0.963 mg/dL and mean red blood cell as well as platelet counts were likely to decrease by factors of 1.352 and 0.966, respectively.

## 5. Conclusions

COVID-19 is a novel infectious disease unprecedented in modern times. Unlike past epidemics, it has resulted in widespread restrictions at the individual, societal, and national levels, with significant global economic impacts. The ongoing nature of the COVID-19 situation, rather than it being a short-term crisis, has underscored the importance of maintaining mental and physical health. The pandemic has necessitated a shift towards reduced in-person interaction, with restrictions on activities such as dining out and social gatherings; however, this study confirms that such lifestyle changes have had negative impacts on physical vital signs and clinical laboratory values.

This study examined the pre- and post-COVID-19 outbreak situations; however, caution is needed in interpreting the following results. A nationwide survey of living conditions was conducted at a time when social and economic facilities were completely suspended or restricted due to the widespread occurrence of the pandemic and national-level disease control measures; however, there are limitations to extrapolating these findings to the direct impact of COVID-19. Additionally, although age, regional, and other stratified surveys were conducted by national research institutions, attention is needed due to the small sample size of this study. Finally, there is a need to directly investigate the impact of COVID-19 on individuals by examining factors such as their physical condition, blood, and urine.

Future research should continue to investigate the impact of COVID-19 on dental practices and explore innovative strategies with which to optimize patient care while ensuring the safety of both patients and dental professionals. Additionally, studies examining the psychological impact of the pandemic on dental professionals and effective interventions to promote their well-being are warranted [41,42].

## Figures and Tables

**Table 1:** General characteristics of the overweight group before and after the COVID-19 outbreak.

Characteristic				Before the COVID-19 Outbreak (2018)		After the COVID-19 Outbreak (2021)		Z ^2^/^4^*X*^2^	*p*-Value ^3^
				N ^5^/Mean ^1^	% ^5^/SD ^1^	N/Mean	%/SD		
Sex ^4^		Male		1817	51.0	1687	51.0	0.002	0.962
		Female		1743	49.0	1622	49.0		
Age ^1^				53.54	15.97	54.73	16.39	−3.382	0.001
Height (cm) ^1^				163.81	9.85	163.97	10.03	−0.501	0.617
Weight (kg) ^1^				70.79	11.58	71.26	12.02	−1.363	0.173
Waist circumference ^1^				88.41	7.95	90.35	8.20	−9.694	<0.001
Body mass index (BMI) ^1^				26.28	2.77	26.39	2.88	−1.499	0.134
Pulse (60 s) ^1^				56.68	10.60	57.62	13.00	−0.711	0.477
Systole ^1, 6^				122.12	16.29	123.32	15.12	−3.593	<0.001
Diastole ^1, 6^				77.39	10.45	75.56	9.60	−8.255	<0.001
BMI ^4^ (weight control for 1 year)	BMI 23.0~24.9 (overweight)		Loss effort	545	39.0	529	41.6	3.908	0.272
			Maintenance effort	313	22.4	299	23.5		
			Gain effort	28	2.0	23	1.8		
			Never effort	510	36.5	420	33.0		
	BMI 25.0~29.9 (obesity)		Loss effort	992	55.3	931	55.8	10.432	0.015
			Maintenance effort	241	13.4	279	16.7		
			Gain effort	6	0.3	5	0.3		
			Never effort	554	30.9	453	27.2		
	BMI 30.0 over (high obesity)		Loss effort	219	64.4	228	65.3	0.249	0.883
			Maintenance effort	32	9.4	35	10.0		
			Gain effort	0	0.0	0	0.0		
			Never effort	89	26.2	86	24.6		

^1^ M: average; SD: standard deviation; ^2^ Mann–Whitney test; ^3^ *p* < 0.05; ^4^ *X*^2^: chi-square test; ^5^ N: frequency; %: percentage; and ^6^ average of two measurements.

**Table 2:** Blood test values before and after the COVID-19 outbreak.

Characteristic		Before the COVID-19 Outbreak (2018)		After the COVID-19 Outbreak (2021)		Z ^2^/^4^*X*^2^	*p*-Value ^3^
		N ^5^/Mean ^1^	% ^5^/SD ^1^	N/Mean	%/SD		
Fasting blood sugar (FBS) ^1^		104.84	24.24	106.60	25.98	−4.508	<0.001
HbA1c ^1^		5.83	0.84	5.97	0.90	−9.984	<0.001
Total cholesterol ^1^		193.81	39.21	189.07	40.17	−4.845	<0.001
HDL cholesterol ^1^		48.02	11.26	49.17	11.66	−4.114	<0.001
Triglycerides ^1^		154.60	118.50	141.33	106.48	−6.643	<0.001
LDL cholesterol ^1^		115.86	33.96	116.24	35.98	−0.075	0.940
Hypercholesterolemia ^4^	No	2370	70.6	2092	66.6	12.557	<0.001
	Yes	985	29.4	1051	33.4		
Hypertriglyceridemia ^4^	No	2233	79.8	2388	84.5	21.389	<0.001
	Yes	566	20.2	438	15.5		
AST(SGOT) ^1^		25.23	14.37	26.41	12.88	−5.977	<0.001
ALT(SGPT) ^1^		26.40	19.20	27.45	21.28	−2.622	0.009
Hepatitis B surface antigen ^4^	Negative	3354	97.1	3141	97.1	0.002	0.966
	Positive	101	2.9	94	2.9		
Hepatitis C antibody ^4^	Negative	3429	99.2	3203	99.0	1.089	0.297
	Positive	26	0.8	32	1.0		
Hemoglobin ^1^		14.37	1.60	14.03	1.58	−8.663	<0.001
Hematocrit ^1^		43.00	4.30	42.56	4.28	−4.074	<0.001
Anemia ^4^	Negative	3234	93.8	2906	89.9	33.283	<0.001
	Positive	215	6.2	326	10.1		
Blood urea nitrogen ^1^		15.74	4.89	15.25	4.73	−4.590	<0.001
Blood creatinine ^1^		0.83	0.21	0.82	0.22	−1.650	0.099
WBC ^1^		6.35	1.74	6.26	1.66	−2.205	0.027
RBC ^1^		4.66	0.50	4.63	0.51	−2.563	0.010
Platelets ^1^		262.32	64.45	253.84	62.79	−5.251	<0.001

^1^ M: average; SD: standard deviation; ^2^ Mann–Whitney test; ^3^ *p* < 0.05; ^4^ *X*^2^: chi-square test; ^5^ N: frequency; and %: percentage.

**Table 3:** Urine test before and after the COVID-19 outbreak.

Characteristic		Before the COVID-19 Outbreak (2018)		After the COVID-19 Outbreak (2021)		Z ^2/4^*X*^2^	*p*-Value ^3^
		N/Mean ^1^	%/SD ^1^	N/Mean	%/SD		
Uric acid ^1^		5.44	1.41	5.47	1.43	−0.970	0.332
Uric acidity ^1^		5.87	0.74	5.90	0.77	−0.940	0.347
Nitrate ^4^	No	3334	97.5	3172	97.7	0.490	0.484
	Yes	87	2.5	74	2.3		
Urine protein ^4^	Negative	2743	80.2	2950	90.9	171.090	<0.001
	Trace	510	14.9	197	6.1		
	1 ^+^	131	3.8	65	2.0		
	2 ^+^	33	1.0	28	0.9		
	3 ^+^	1	0.0	4	0.1		
	4 ^+^	3	0.1	2	0.1		
Urine glucose ^4^	Negative	3241	94.7	2984	91.9	40.045	<0.001
	Trace	44	1.3	66	2.0		
	1 ^+^	23	0.7	35	1.1		
	2 ^+^	33	1.0	25	0.8		
	3 ^+^	38	1.1	33	1.0		
	4 ^+^	42	1.2	103	3.2		
Urine ketone ^4^	Negative	3370	98.5	3209	98.9	20.122	<0.001
	Trace	17	0.5	0	0.0		
	1 ^+^	20	0.6	28	0.9		
	2 ^+^	12	0.4	9	0.3		
	3 ^+^	2	0.1	-	-		
	4 ^+^	-	-	-	-		
Urine bilirubin ^4^	Negative	3397	99.3	3246	100.0	22.855	<0.001
	trace	-	-	-	-		
	1 ^+^	24	0.7	-	-		
	2 ^+^	-	-	-	-		
	3 ^+^	-	-	-	-		
	4 ^+^	-	-	-	-		
Urine occult blood ^4^	Negative	2831	82.8	3034	93.5	191.933	<0.001
	trace	336	9.8	122	3.8		
	1 ^+^	138	4.0	32	1.0		
	2 ^+^	67	2.0	30	0.9		
	3 ^+^	43	1.3	28	0.9		
	4 ^+^	6	0.2	-	-		
Urine bilinogen ^4^	Negative	3405	99.5	3222	99.3	8.533	0.014
	trace	4	0.1	-	-		
	1 ^+^	12	0.4	23	0.7		
	2 ^+^	-	-	1	0.0		
	3 ^+^	-	-	-	-		
	4 ^+^	-	-	-	-		
Urine creatinine ^1^		147.08	80.10	125.89	74.77	−11.726	0.000
Urine sodium ^1^		116.79	48.09	113.66	47.75	−3.212	0.001
Urine potassium ^1^		52.66	23.22	41.51	20.75	−19.034	0.000
Urine cotinine ^1^		346.22	718.56	783.69	826.45	−23.264	0.000

^1^ M: average; SD: standard deviation; ^2^ Mann–Whitney test; ^3^ *p* < 0.05; ^4^ *X*^2^: chi-square test; ^5^ N: frequency; %: percentage.

**Table 4:** Blood and urine test values before and after the COVID-19 outbreak.

Blood Test *					Urine Test *				
	ORs	95% CI		*p*-Value		ORs	95% CI		*p*-Value
Systole	1 + 0.019	1.014	1.024	<0.001	Systole	1.018	1.010	1.025	<0.001
					Diastole	0.958	0.948	0.969	< 0.001
Diastole	0.964	0.956	0.972	<0.001	Protein No				<0.001
					Ttrace	0.385	0.276	0.537	<0.001
FBS	1.000	0.995	1.004	0.910	Protein ^1+^	0.597	0.340	1.049	0.073
					Protein ^2+^	1.281	0.487	3.371	0.616
HbA1c	1.062	0.934	1.206	0.359	Protein ^3+^	5.83 × 10^9^	0.000		0.999
Total cholesterol	0.999	0.997	1.001	0.201	Protein ^4+^	0.000	0.000		0.999
					Glucose No				0.001
HDL cholesterol	1.010	1.004	1.016	0.001	Trace	1.832	0.980	3.425	0.058
					Glucose ^1+^	1.949	0.807	4.710	0.138
Triglycerides	1.000	0.999	1.000	0.322	Glucose ^2+^	0.420	0.171	1.028	0.057
					Glucose ^3+^	0.630	0.280	1.415	0.263
Hypercholesterolemia, yes	1.131	0.993	1.287	0.063	Glucose ^4+^	2.311	1.350	3.955	0.002
					Ketone No				0.699
Hypertriglyceridemia, yes	0.917	0.741	1.134	0.422	Trace	0.000	0.000		0.998
					Ketone ^1+^	1.460	0.556	3.829	0.442
AST (SGOT)	1.004	0.998	1.011	0.189	Ketone ^2+^	1.861	0.498	6.945	0.355
					Urine bilirubin Trace	0.000	0.000		0.999
ALT (SGPT)	1.003	0.998	1.008	0.245	Urine occult blood No				<0.001
					Trace	0.374	0.246	0.568	<0.001
Hemoglobin	0.280	0.240	0.326	<0.001	Occult blood ^1+^	0.203	0.085	0.481	<0.001
					Occult blood ^2+^	0.477	0.193	1.178	0.108
Hematocrit	1.476	1.386	1.572	<0.001	Occult blood ^3+^	0.963	0.408	2.274	0.931
					Occult blood ^4+^	0.000	0.000		0.999
Blood urea nitrogen	0.963	0.950	0.975	<0.001	Urobilinogen No	-	-	-	0.660
					Trace	0.000	0.000	-	0.999
WBC	0.964	0.928	1.001	0.056	Urobilinogen ^1+^	1.584	0.589	4.261	0.362
					Urine creatinine	1.003	1.001	1.005	<0.001
RBC	1.352	1.016	1.801	0.039	Urine sodium	1.000	0.998	1.002	0.776
					Urine potassium	0.973	0.968	0.977	<0.001
Platelets	0.996	0.995	0.997	<0.001	Urine cotinine	1.001	1.001	1.001	<0.001

^1^ M: average; SD: standard deviation; ^2^ Mann–Whitney test; ^3^ *p* < 0.05; ^4^ *X*^2^: chi-square test; ^5^ N: frequency; and %: percentage. * Multi-variable regression analysis adjusted for age, waist circumference, and BMI.

## Data Availability

Data are contained within the article.

## References

[1] Afonso P. (2020). The impact of the COVID-19 pandemic on mental health. Acta Medica Port..

[2] Ministry of Culture, Sports and Tourism (2020). Public Perception Survey Regarding COVID-19 Self-Isolation. http://www.mcst.go.kr/english/.

[3] Park M.G. (2020). Corona 19 Crisis’ and Seeking Socio-Economic Transformation. Labor Rev..

[4] Korea Disease Control and Prevention Agency (2020). Press Release. https://www.kdca.go.kr/board/board.es?mid=a30402000000&bid=0030.

[5] Kim E.Y., Kim J.Y. (2022). Adult’s Perception of Every Day Life Change After COVID-19. J. Korea Acad.-Ind. Coop. Soc..

[6] Rotter M., Brandmaier S., Prehn C., Adam J., Rabstein S. (2017). Stability of targeted metabolite profiles of urine samples under different storage conditions. Metabolomics.

[7] Ministry of Health and Welfare (2020). (2.23) Briefing on the Pan-Governmental Meeting for COVID-19. https://www.mohw.go.kr/board.es?mid=a20401000000&bid=0032&tag=&act=view&list_no=353124.

[8] World Health Organization (2021). WHO Coronavirus (COVID-19) Dashboard.

[9] Park S.M. (2020). The impact of the COVID-19 pandemic on mental health among population. J. Health Educ. Promot..

[10] Ministry of Health and Welfare (2020). COVID-19 Pan-Government Preparedness Conference Briefing.

[11] Jung C.H., Son J.W., Kang S., Kim W.J., Kim H.S., Kim H.S., Seo M., Shin H.-J., Lee S.-S., Jeong S.J. (2021). Diabetes fact sheets in Korea, 2020: An appraisal of current status. J. Diabetes Metab..

[12] Korean Diabetes Association (2020). Diabetes Fact Sheet in Korea.

[13] Kim H.S. (2019). Importance of Target Blood Pressure Management in Diabetic Kidney Disease. J. Korea Contents Assoc..

[14] Kim J.Y. (2014). The Study of Physical Activity Level on Serum BDNF and Cognitive Function in Adolescence. J. Growth Dev..

[15] Oh J.W., Woo S.S., Kwon H.J., Kim Y.S. (2013). Examining the Association of Physical Activity and PAPS Health-related Physical Fitness on the Physical Self-Description of a Specialized Male Highschool Students. J. Phys. Educ..

[16] Lee B.S. (2004). A Comparative Study on Dietary Life and Recognition of Diet Related Factors in Elementary, Middle and High School Students. Korean J. Diet. Assoc..

[17] Lee A., Tsang C.K. (2004). Youth risk behavior in a Chinese population: A territory wide youth risk behavioral surveillance in Hong Kong. J. Public Health.

[18] Lee Y.J., Kim J.H. (2022). A Study Analyzing the Relationship among Impaired Fasting Glucose (IFG), Obesity Index, Physical Activity, and Beverage and Alcohol Consumption Frequency in 20s and 30s: The Korea National Health and Nutrition Examination Survey (KNHANES) 2013–2015. J. Community Living Sci..

[19] Korea Diabetes Association (KDA) (2021). Clinical Practice Guidelines for Diabetes.

[20] Blair S.N., Broney S. (1999). Effects of physical inactivity and obesity on morbidity and mortality: Current evidence and research issues. Med. Sci. Sports Exerc..

[21] Jekal Y., Lee M.K., Kim E.S., Park J.H., Lee H.J., Han S.J., Kang E.S., Lee H.C., Kim S.Y., Jeon J.Y. (2008). Effects of walking and physical activity on glucose regulation among type 2 diabetics. J. Korean Diabetes.

[22] Singh G.M., Micha R., Khatibzadeh S., Lim S., Ezzati M., Mozaffarian D. (2015). Chronic Diseases Expert G (2015) Estimated global, regional, and national disease burdens related to sugarsweetened beverage consumption in 2010. Circulation.

[23] Kim Y., Park S., Oh K., Choi H., Jeong E.K. (2023). Changes in the management of hypertension, diabetes mellitus, and hypercholesterolemia in Korean adults before and during the coronavirus disease 2019 pandemic: Data from the 2010–2020 Korea National Health and Nutrition Examination Survey. Epidemiol. Health.

[24] Han I.H., Chong M.Y. (2020). The Study on the Difference of Blood Level of HDL-Cholesterol by Obesity and Health Behavior from the Seventh (2016) Korea National Health and Nutrition Examination Survey. Korean Soc. Food Sci. Nutr..

[25] Committee for Guidelines for Management of Dyslipidemia (2015). Korean guidelines for management of dyslipidemia. J. Lipid Atheroscler..

[26] Dichtl W., Nilsson L., Goncalves I., Ares M., Banfi C., Calara F., Hamsten A., Erilsson P., Nilsson J. (1999). Very low-density lipoprotein activates nuclear factor-κB in endothelial cells. Circ. Res..

[27] Lutgens E., Van Suylen R.J., Faber B.C., Gijbels M.J., Eurlings P.M., Bijnens A.P., Cleutjens K.B., Heeneman S., Daemen M. (2003). Atherosclerotic plaque rupture: Local or systemic process?. Arterioscler. Thromb. Vasc. Biol..

[28] Gordon T., Castelli W.P., Hjortland M.C., Kannel W.B., Dawber T.R. (1977). High density lipoprotein as a protective factor against coronary heart disease. The Framingham Study. Am. J. Med..

[29] Song S.O., Hwang Y.C., Kahn S.E., Leonetti D.L., Fujimoto W.Y., Boyko E.J. (2020). Intra-abdominal fat and high density lipoprotein cholesterol are associated in a non-linear pattern in Japanese Americans. Diabetes Metab. J..

[30] de Melo L.G.P., Nunes S.O.V., Anderson G., Vargas H.O., Barbosa D.S., Galecki P., Carvalho A.F., Maes M. (2017). Shared metabolic and immune-inflammatory, oxidative and nitrosative stress pathways in the metabolic syndrome and mood disorders. Prog. Neuro-Psychopharmacol. Biol. Psychiatry.

[31] Atti A.R., Valente S., Iodice A., Caramella I., Ferrari B., Albert U., Mandelli L., De Ronchi D. (2019). Metabolic syndrome, mild cognitive impairment, and dementia: A meta-analysis of longitudinal studies. Am. J. Geriatr. Psychiatry.

[32] Jin S.H. (2019). The Relation of Impaired Fasting Glucose and HDL-Cholesterol by Gender and Body Mass Index. J. Health Inform. Stat..

[33] Pal K., Mukadam N., Petersen I., Cooper C. (2018). Mild cognitive impairment and progression to dementia in people with diabetes, prediabetes and metabolic syndrome: A systematic review and meta-analysis. Soc. Psychiatry Psychiatr. Epidemiol..

[34] Rashid S., Genest J. (2007). Effect of obesity on high-density lipoprotein metabolism. Obesity.

[35] Seo J.B., Chung W.Y. (2008). The importance of treatment of low HDL cholesterolemia in cardiovascular disease. J. Lipid Atheroscler..

[36] Dufour D.R., Lott J.A., Nolte F.S., Gretch D.R., Koff R.S., Seeff L.B. (2000). Diagnosis and monitoring of hepatic injury. II. recommendations for use of laboratory tests in screening, diagnosis, and monitoring. Clin. Chem..

[37] Weiss G., Goodnough L.T. (2005). Anemia of chronic disease. N. Engl. J. Med..

[38] Sharrett A.R., Ballantyne C.M., Coady S.A., Heiss G., Sorlie P.D., Catellier D., Patsch W. (2001). Coronary heart disease prediction from lipoprotein cholesterol levels, triglycerides, lipoprotein(a), apolipoproteins A-I and B, and HDL density subfractions: The Atherosclerosis Risk in Communities (ARIC) study. Circulation.

[39] Cavezzi A., Emidio T., Salvatore C. (2020). COVID-19: Hemoglobin, iron, and hypoxia beyond inflammation. A narrative review. Clin. Pract..

[40] Medical Information on the National Health Information Portal. https://health.kdca.go.kr/healthinfo/.

[41] Paolone G., Mazzitelli C., Formiga S., Kaitsas F., Breschi L., Mazzoni A., Tete G., Polizzi E., Gherlone E., Cantatore G. (2022). One-year impact of COVID-19 pandemic on Italian dental professionals: A cross-sectional survey. Minerva Dent. Oral. Sci..

[42] Gherlone E., Polizzi E., Tete G., Cappare P. (2021). Dentistry and Covid-19 pandemic: Operative indications post-lockdown. New Microbiol..

